# Assessing value in health care: using an interpretive classification system to understand existing practices based on a systematic review

**DOI:** 10.1186/s12913-019-4405-6

**Published:** 2019-08-13

**Authors:** Brayan V. Seixas, François Dionne, Tania Conte, Craig Mitton

**Affiliations:** 1 Department of Health Policy and Management, Fielding School of Public Health, University of California, Los Angeles (UCLA), Los Angeles, USA; 2 Prioritize Consulting, Vancouver, Canada; 3 Center for Clinical Epidemiology and Evaluation, Vancouver, Canada; 4 School of Population and Public Health, University of British Columbia (UBC), Vancouver, Canada

**Keywords:** Value assessment, Frameworks, Resource allocation, Efficiency

## Abstract

**Background:**

Implementing adequate strategies to assess the value of health services plays a central role in the effort to deal with the financial pressures faced by health care systems worldwide. This study aimed to understand which approaches to value assessment have been used in developed countries.

**Methods:**

We conducted a rapid review and a gray literature search to identify value assessment frameworks. A two-stage screening process was utilized to identify existing approaches and cluster similar frameworks. In addition, we developed an interpretive classification system to make sense of existing approaches.

**Results:**

One thousand one hundred seventy-six references were identified and 38 papers were selected for full-review. Among these 38 articles, 22 distinct approaches to assess value of health care interventions were identified and classified according to four points: 1) use of single or multiple considerations to base value estimates; 2) use of disease-specific or generic criteria; 3) reliance on process-based or outcomes-based consideration; and 4) type of input and evidence considered.

**Conclusions:**

The contextual nature of value assessment in health care becomes evident with the diversity of existing approaches. Despite the predominance of cases relying on the Incremental cost-effectiveness ratio as the measure of value, this approach has not been sufficient to meet the needs of decision-makers. The use of multiple criteria has become more and more important, as well as the consideration of patient-reported measures. Considerations of costs are not always explicit and consistent.

**Electronic supplementary material:**

The online version of this article (10.1186/s12913-019-4405-6) contains supplementary material, which is available to authorized users.

## Background

The unsustainable growth of health expenditures observed in developed countries over the last decades has sparked a variety of initiatives worldwide envisioning adequate allocations of the scarce resources available. Economic evaluations of care interventions, experiments involving payment schemes, campaigns for appropriateness of care targeting clinicians, novel purchasing strategies, among so many others, there seem to exist initiatives targeting virtually every aspect of the health care system. In recent years, the term value has occupied a central spot in this constellation of efforts toward wiser spending of money.

Assessing the value (often referred to as the ‘value for money’) of health care technologies, in particular the novel ones, has received an enormous attention from researchers, policy-makers and the industry. In countries with single-payer systems, the economic evaluation of technologies acquired a paradigmatic status, although its practice has been often incomplete or inconsistent. In the United States, where co-exist a market-oriented system of private insurers/providers and public programs (distinct among themselves, such as Medicare, Medicaid and the VA system), the notion of value has been yet incorporated but it has gained more and more attention in the latest years, despite the unclear lieu of value-based strategies in general.

Notwithstanding the disseminated use of the term ‘value’, it has not always been employed with the same meaning. The most commonly accepted definition of value in health care is provided by Michael Porter as “health outcomes achieved per dollar spent” [1]. This description is conceptually situated within the realm of technical efficiency, concerning about the maximization of objective gains in health in relation to a given amount of financial resources. In defining value at the ISPOR Special Task Force Report, in turn, Garrison et al. point out that “from an economic perspective: the ‘gross value’ can be thought of what someone would be willing to pay for an economic good or intervention, whereas the ‘net value’ subtracts the opportunity cost incurred to obtain that gross value” [2], p124). In the latter definition, on contrary, value evocates a connotation of allocative efficiency.

Although Garrison et al. argue that the underpinning notion of value does not depend on the nature of the health care system (whether a market-based, a social insurance or a single-payer system), that might be true from a normative perspective, but it does not seem the case considering the diversity of ways the term value is employed and the distinct approaches it motivates. The corollary of this observation is that, apart from the unsurprising idea that the value of health care technologies is contextual for many reasons (the perspectives included in the assessment, the societal values, the relative price of labor and technology, etc.), the proper concept of value seems to be contextually sensitive.

The aim of the present study was to understand what strategies of value assessment have been developed and implemented worldwide. In addition, we sought to comprehend the contexts in which these initiatives emerged and what definition of value underpinned it. In order to address these research questions, we performed a rapid literature review and a gray literature search focusing on existing value assessment frameworks of health care technologies within the context of developed countries.

## Methods

### Two-stage screening scientific literature review

A comprehensive search of the peer-reviewed literature published between 2007 and 2017 was conducted using Ovid MEDLINE, an extensive database of public health journals with a powerful platform for building searching strategies. The specific search strategy, presented in Additional file 1, resulted in the identification of 1176 references. Following execution of this search, we used a two-stage screening process.

The first stage consisted of selecting articles for full-review and the grouping of similar approaches in preliminary heuristic categories. Titles and abstracts were screened with the following two inclusion criteria: 1) does the paper describe a way to assess value of a treatment, service, intervention or technology? And 2) does the paper describe an applied framework (i.e., was the value assessment approach actually implemented)? In addition, we excluded papers that: 1) deal with animal health; 2) describe means to assess the value of management systems, administrative procedures, payment schemes, data management solutions, training programs, human resources schemes, or solutions for health care supply chains and purchasing contracts; or 3) that are not full articles (i.e., editorial, interview, commentary).

All 1176 titles/abstracts were reviewed by one reviewer and 335/1176 (28%) were also reviewed by a second reviewer. The agreement rate between the two reviewers on the first screening was over 95%, with discrepancies settled by discussion. There were no cases where a third reviewer was required. In total, 157/ 1176 papers were initially screened ‘in’ for further review. In order to make sense of the data and help in the understanding of the big picture, each of these 157 references were placed into one of six initial descriptive categories: a) CEA/CUA; b) simple consideration of costs and outcomes; c) unspecified value approach; d) multiple criteria approach; e) net economic/social value; and f) comparative effectiveness.

The second stage of screening was meant to further refine and hone the initial six categories listed above. In this stage, we were working to saturation, so we sought to include papers that described empirical activity of approaches to value assessment while excluding papers there were simply ‘more of the same’. In addition, we sought to identify any particularly innovative or novel aspects in the implementation of a given approach. This second screening resulted in the selection of 38 articles to which we applied our data extraction tool (see Additional file 2) for classification and evaluation of value assessment approaches.

### Gray literature search

To complement our peer review literature search, we also conducted a gray literature search. Two main strategies of environmental scan were used. First, we relied on previous systematic reviews of initiatives around disinvestment and/or reassessment of low-value and/or potentially obsolete health technologies to ensure that we are getting at value in the context of resource management [3–8]. A search was conducted on the websites of institutions found in these reviews (see Additional file 3). The terms used for the search on each of the identified websites were: *low value, disinvestment, reassessment, de-adoption, decommissioning and delisting*. The second search strategy consisted of exploring the websites of highly reputable HTA agencies and other relevant professional organizations for presentations, guidelines, working papers or any other pertinent piece of gray literature. For this purpose, we looked at the following organizations: Health Technology Assessment International (HTAi); International Network of Agencies for Health Technology Assessment (INAHTA); International Society for Pharmacoeconomics and Outcomes Research (ISPOR); European Network for Health Technology Assessment (EUnetHTA); International Health Economics Association (IHEA); Agency for Health Research and Quality (AHRQ); Canadian Agency for Drugs and Technologies in Health (CADTH); Kaiser International Health Group; and Blue Cross Blue Shield Association. A total of 1390 documents were identified, of which 52 qualified for full review.

## Results

**Fig. 1:**
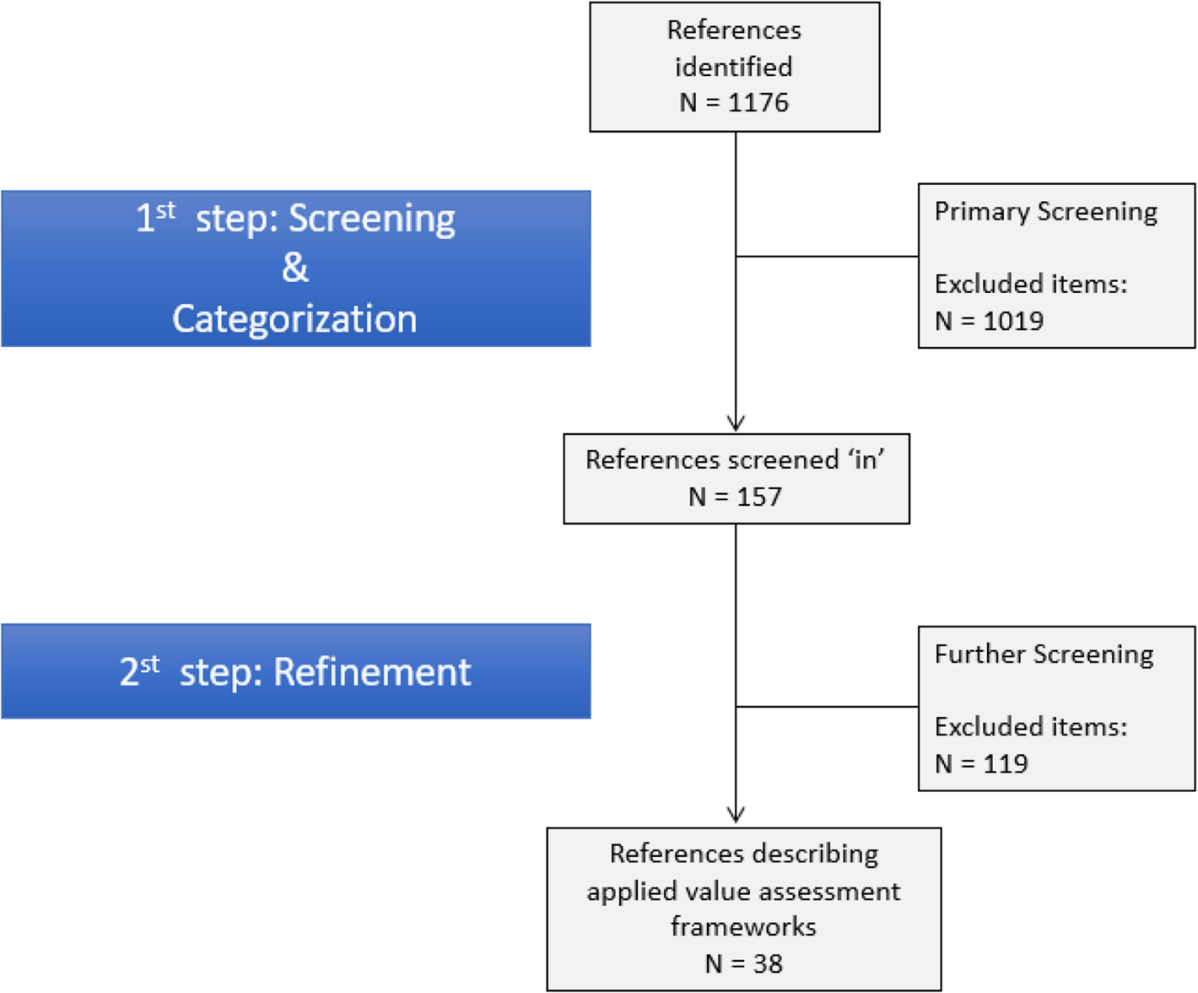
Literature search process and results

Figure 1 presents the two-stage screening process. We found 22 distinct approaches to assess the value of health care interventions, which were reported in the 38 papers from the peer-reviewed literature search. The gray literature search returned only methodologies and discussions on processes to identify and prioritize low value or obsolete technologies for further evaluation but no particular approach to assess their value per se was identified.

No gold standard value assessment framework was reported despite the supposedly theoretical supremacy of cost-effectiveness analysis. The existing strategies found here are diverse and vary according to their context and specific objectives. Given the large and heterogenous number of frameworks, our narrative synthesis process involved the development of a classification system to categorize the methodologies found. Our analytical scheme is based on the key structuring questions observed across the existing frameworks of value assessment.

The classification we propose is built around four questions: 1) Is the method based on one consideration/criterion or multiple considerations/criteria? 2) Are the considerations/criteria disease-specific or more generic? 3) Are the considerations/criteria process-oriented or are they directly based on patients’ perspective? 4) What input is primarily used to measure performance and what is the acceptable ‘evidence’?

These four questions expand on the two fundamental issues that must be addressed in any value assessment exercise: what is the entity to be measured and how is it to be measured.

### Question #1: one consideration or multiple considerations?

Based on the nature of the health care service to be assessed, one has to determine the relevant criteria to guide decisions. Most scenarios, if not all, are complex and involve several components and objectives. However, it is not always feasible and practical to take into consideration all relevant aspects pertaining to an intervention. In these cases, the use of ‘one consideration’ seems the most common choice, having cost-effectiveness/cost-utility analysis (CEA/CUA) as the most prevalent framework. There is a broad evidence base supporting the use of CEA in assessing value. It should be noted that with CEA/CUA, outcomes can be defined in many different ways, from life years gained to symptoms avoided to units of clinical benefit. On the other hand, if multiple considerations are deemed relevant, then a myriad of approaches may be employed. Although the majority of the value assessment frameworks falling within the multiple consideration category draw some elements from the set of tools broadly known as multi-criteria decision analysis (MCDA), it is rare to see a consistent, structured and explicit discussion of these theoretical elements of decision sciences.

It is important to explain the choice of using the term ‘consideration’ instead of ‘criterion’ or ‘criteria’. Even though ‘criteria’ is indeed an appropriate term for most of the value assessment frameworks identified here, which basically consist of judging an intervention according to a principle, we found that the term is not entirely suitable for some approaches. Thus, the word ‘consideration’ was chosen as a way to avoid semantic inadequacies in some cases, but these terms can be used interchangeably.

### Question #2: disease-specific or more generic consideration?

The key in addressing this question is determining whether or not there is a need for generalizability across a range of interventions. If the need for a particular assessment of value is for the purpose of informing choices between similar interventions, then disease-specific criteria are preferable because they will by nature be more directly relevant and the results will typically be more precise. If the decision-making applies to a broader context across different areas of care and levels of organization, then more generic criteria are more appropriate. From an economic perspective, this question relates to whether the decision at hand is one of technical efficiency (i.e., given a decision to treat a certain patient group, what is the best treatment option) or of allocative efficiency (i.e., how to allocate resources among interventions aiming different patient groups).

### Question #3: process-based or patient-oriented outcomes?

This question relates to the point in the health care system where value is assessed. If one is looking at the entire service continuum, then a patient outcomes perspective seems intuitive. On the other hand, if the focus is on a specific element within that continuum, such as rehabilitation, or post-surgical care, then adherence to clinical guidelines might be a more appropriate way to assess value as it attempts to isolate the given intervention from the influence of other parts of care. Patient reported outcomes have been around for many years but recently have received greater attention across all health systems.

### Question #4: what input and what ‘evidence’?

This question refers to the nature of information used to address the previous questions and the types of evidence acceptable to base these answers. The answer to this fourth question depends in part on the choices made in questions two and three. For example, process-oriented considerations are more likely going to be data-driven whereas certain health outcomes might be possibly best assessed by expert opinion, including, where possible, patients’ feedback. In addition, disease specific contexts are more likely to have established measurement tools. In contrast, when looking at value assessment across disease areas there is likely only lower level evidence available as there are going to be fewer ‘head to head’ studies of disparate treatment areas. In these cases, expert opinion may probably come to play a role in the assessment.

### Classification summary

In order to illustrate how this classification might work, take for example the well-known ASCO framework. In this case, for the first question, benefit gains are measured against more than one consideration; for the second question, the focus is on cancer so the criteria lean more towards disease-specific than generic, without being so specific as to apply to only one cancer; for the third question, the criteria focus on health outcomes; and finally, for the fourth question, input is from clinical trial results with the addition of expert opinion, as necessary. Table 1 presents the 22 approaches organized as per our classification system. It must be noted that some of the approaches are, by their nature, very specific in their content while others are made to adjust to the context where they are applied. For example, the ASCO framework contains specific criteria with specific weights while ‘MCDA’ – as a more generic approach - covers any set of criteria developed for any given implementation.

**Table 1:** Classification of existing value assessment frameworks

	Contextual information		Interpretive classification system			
Approach Name	Who uses it?	Which purpose?	One consideration or multiple considerations?	Disease-specific or generic criteria?	Process-related or outcomes criteria?	Who inputs and what evidence?
American Society of Clinical Oncology (ASCO) framework [9, 10]	The American Society of Clinical Oncology	To provide a formal approach to define value of cancer treatments and a tool to facilitate one-on-one discussions with patients regarding the relative value of various treatment options.	Multiple	Specific	Outcomes	Trials; expert panel; patient feedback
Comparative effectiveness Research [11, 12]	- Pharmaceutical industry clinical trial professionals - Health Maintenance Organizations (HMOs) - Veterans Health Administration	- To determine the intervention with the best value for that specific disease. - To discuss the challenges and opportunities to develop comparative research and using its results to improve patient care.	Multiple	Specific	Outcomes	Data-driven
Cost/Value Methodology [13]	- American College of Cardiology - American Heart Association	- To enhance overall value in the delivery of cardiovascular care. - To involve healthcare professionals in the difficult decisions that must be made to increase value in the U.S. healthcare system.	One	Generic	Outcomes	Clinical trials
Cost-effectiveness / Cost-utility Analysis [14–25]	- Health care centers - Academic researchers - HTA agencies - Governmental departments responsible for health care funding - Pharmaceutical industries	- To determine the more effective and economically attractive strategy among a set of options - To assess whether specific technology is cost-effective in comparison to mainstream intervention	One	Generic	Outcomes	Clinical trials/ decision analysis
Discrete Choice Experiment (DCE) [26]	- International collaboration of academic researchers	To elicit social values and public preference for the allocation of resources across a wide range of health technologies	Multiple	Generic	Outcomes	Survey of public
Drug Abacus framework [27]	- Drug Abacus	To assess the value of cancer drugs, allowing users to build preferences for different drug attributes into the tool’s value.	Multiple	Generic	Outcomes	Expert opinion
Economic priority and conformity [28]	- Academic researchers in Australia	To establish the value of Indigenous eye health programs (IEHPs) using not only the health and heath care needs approach, but also the economic priority and performance standards approach using all relevant benchmarks.	Multiple	Specific	Outcomes	Data-driven
High value care process [29]	Division of Trauma and Critical Care, Department of Surgery, Cedars-Sinai Medical Center, Los Angeles, California	To optimize the use of ancillary services in ICU.	Multiple	Specific	Process and outcomes	Data-driven
Institute for Clinical and Economic Review (ICER) framework [27, 30]	Institute for Clinical and Economic Review framework	To assess the value of cancer drugs.	Multiple	Generic	Outcomes	Clinical trials and expert opinion
Multi-criteria Decision Analysis (MCDA) [31, 32]	- Health care organizations - Private companies focused on evidence-based assessments. - Academic researchers - International Society for Pharmacoeconomics and Outcomes Research (ISPOR) Task Force	- To provide transparency and consistency for decision making processes. - To combine multiple criteria in a single judgment of a health care technology by multiple stakeholders.	Multiple	Generic	Outcomes	Expert panel
National Comprehensive Cancer Network (NCCN) framework [30]	National Comprehensive Cancer Network	To assess the value of drug treatments with the consideration of multiple criteria.	Multiple	Specific	Outcomes	Expert opinion
Net economic return [33, 34]	- Academic researchers	- To assess whether the value of changes in health care for patients with type 2 diabetes, defined as the prevention of future mortality and morbidity, exceeds the increase in costs of that management. - To estimate the clinical and economic return of trial compared to a scenario where the trial had not been conducted.	One	Generic	Outcomes	Data-driven
Program Budgeting and Marginal Analysis (PBMA) [35–37]	- Academic researchers - Health care organizations - Governmental health care authorities	- To serve as a broad mean of prioritizing resource allocation. - To improve the management of public health interventions at the national level (in Wales), bringing a culture of evidence-based decision making into routine policy. - To bring expenditure in line with available funds. - To determine a list of options for disinvestment generating cost savings for other investment intentions.	Multiple	Generic	Outcomes	Expert panel
Six Sigma methodology [38]	Hospital	- To explore the waste location in a process and to identify the risk in advance and prevent the occurrence of possible errors in the process. - To reach the goal of “zero” specimen rejections within the hospital.	Multiple	Specific	Process	Data-driven
Surgical auditing [39]	Hospital	To provide/improve quality information leading to the identification of existing problems in the care process.	Multiple	Specific	Outcomes	Data-driven
System cost-effectiveness [40]	- Academic researchers -Health care organizations	To elicit the health value of suboptimal treatment approaches.	Multiple	Specific	Outcomes	Expert panel
Systematic evidence-based quality measurement [41]	- Centers for Medicare and Medicaid Services (CMS) - Agency for Healthcare Research and Quality (AHRQ)	To reexamine selected health care quality measures from a child core set (CCS) voluntarily reported on by a number of state Medicaid and Children’s Health Insurance Program (CHIP) programs over the 3 federal fiscal years in the US.	Multiple	Specific	Process	Data-driven
Value framework for specialty drugs [42]	Hospital Pharmacy & Therapeutics Committee	To assess the value of specialty drugs in order to decide which ones to be funded within a hospital setting.	Multiple	Generic	Outcomes	Expert opinion
Value-based decision [43]	Hospital	To determine the relevance, quality, and cost of perioperative clinical initiatives.	One	Specific	Process	Data from monitoring
Value-based proposition [44]	Academic researchers	To test funding propositions based on Porter’s model of value	Multiple	Specific	Outcomes	Expert opinion
Value-driven outcomes [45]	Academic researchers in the US	To understand and improve healthcare value that is focused on delivering practical utility (pragmatic), implemented using components that can be independently enhanced (modular), and capable of being improved over time (extensible).	Multiple	Specific	Process	Data-driven
Value-driven outcomes program [46]	Academic researchers in the US	- To identify overall care costs across the health care system. - To measure cost variability across Medicare severity diagnosis related groups (MS-DRGs) to identify the greatest opportunities for cost reduction and outcome optimization. - To support value improvement initiatives for selected conditions.	Multiple	Generic	Outcomes	PROMs

Our classification is not making any normative statements, rather it is meant as a guide to help in understanding the myriad of value assessment approaches that are currently reported in the literature. Using the four questions to classify the 22 approaches that were identified in the peer-reviewed literature we found that 18/22 use multiple criteria, 12/22 use criteria that are more disease-specific in nature, 17/22 focus on outcomes-oriented criteria and 12/22 have performance measurements that are mostly data-driven.

In addition, explicit process evaluation took place in about one third of the 38 papers reporting on the various approaches. Key evaluation findings across these studies included the need for transparency in both criteria and methods, the relevance of expert panels to support and contextualize data, and in many (but not all) cases, the application of the value assessment approach was found to lead to changes either in resource use or clinical outcomes.

## Discussion

All studies found in the literature review identify the depicted methodologies as strategies or frameworks for assessing value of health care technologies. However, only a few explicitly state their underlying concept of value. Some articles that report cost-utility analysis agree with Porter defining value broadly as “health outcomes obtained per dollar spent”. Govaert et al. [39] provide a similar but more specific definition of value in a paper on surgical auditing, understanding value as “the health outcomes achieved that matter to patients, relative to costs of achieving those outcomes”. In the DCE experiment conducted by Green and Gerard [26], they sought to estimate the ‘social value’ of health care technologies, in which construct the notion of ‘value for money expected from the treatment’ is a component. It can be said that it represents an attempt to obtain a measure of allocative efficiency that rely on individuals’ judgement on the evidence around technical efficiency. ASCO defines value “as a combination of clinical benefit, side effects, and improvement in patient symptoms or quality of life in the context of cost”.

Despite the lack of a consensual definition of value and the diversity of frameworks as well as contexts where these emerge, we are able to identify two main constructs across the existing initiatives that represent the key challenges in measuring what is achieved by an intervention: what are the outcomes that are to be considered and how is the level of each outcome measured. We expect that each condition will have a unique set of relevant outcomes and, for any condition, the relative importance of each outcome may vary across patient groups. For example, some patient groups may be more risk averse and for those, the rate of adverse events is a more important outcome than to less risk adverse patients. A further complication in the assessment of the value of an intervention is that, when the focus is on outcomes, the contribution of any specific intervention depends in part on the effectiveness of other interventions related to the condition being addressed [1]. These challenges mean that, as we investigate ways to assess the value of health care, we do not expect that there is only one correct ‘value’ for any intervention, even for a specific condition, i.e. ‘value’ is contextual. Our review reveals the inexistence of a gold standard approach, as the right answer is predicated on the particular context and decision that needs to be addressed.

Seeking to understand the current state of affairs in the realm value assessment, we examined both the peer-reviewed literature and gray literature. These reviews focused on actual cases of value measurement as opposed to theoretical frameworks. It is possible that powerful insights might be obtained from theoretical strategies not covered here. Another limitation of our study is that the search was constrained to the developed world and we acknowledge that interesting initiatives may be taking place elsewhere. And it is also possible that existing value assessment frameworks that are currently used have not been published. In order to address this weakness of a sole focus on the peer-reviewed literature, we also included a gray literature search, seeking to capture broader sources of information.

The fact that we were able to identify 22 different approaches in the peer reviewed literature supports the Sorenson et al. [47] conclusion that there is no single approach that is likely to apply in most situations: “At this time, it is clear that there is no one perfect model or framework for value assessment, or even one that will garner consensus across all stakeholders.” So, what can we learn from these 22 approaches found in our review? A few clear messages emerge.

First, there seems to be an awareness across developed countries, and very much so in the US, of the need to improve the measurement of the value of health care services and there obviously have been serious efforts made to do so. That being said, while the convergence in the results of the different approaches seems to be growing [30], these approaches all seem to have some weaknesses. For example, as Cohen et al. [27] state, “all of the frameworks suffer from varying degrees of arbitrariness, namely, subjectively determined end-points and arbitrary ways of combining scores from multiple dimensions to arrive at a composite health outcome measure. Owing to this arbitrariness, it is unknown the degree to which these frameworks capture value accurately.” The awareness of the need to measure value is related to the financial pressures present in health care. This was confirmed in our gray literature search where we did not find any *new* approach to value measurement but where we found many instances of efforts to apply value measurement to the search for interventions that should be avoided because they provide low value for their cost. Furthermore, this perhaps speaks to the notion that the *practice* of value assessment is less developed than the writing and publishing on this subject.

The second message is that while the most prevalent approach cited in the literature is the single criterion CEA/CUA, the vast majority of the 22 approaches found utilize multiple criteria, perhaps reflecting the reality that decision makers typically face a broad set of objectives when assessing value.

Third, the accepted forms of evidence are expanding from the traditional gold standard level of evidence (i.e., RCTs). In fact, about a third of the approaches considered explicitly patient input as part of the process for determining value. This certainly fits with broader trends towards patient-oriented research and the inclusion of patient input in health care decision making [37, 48].

And finally, the focus of most approaches seems to be directly on health outcomes rather than on process measures. The fact that some approaches include disease specific criteria while others are more generic in nature likely reflects decision making practice, i.e., in some cases the focus of the question being addressed is narrower while in other cases the question is broader.

While a comprehensive view of existing approaches for assessing value may be useful to provide insights on further action, the question of what approach should be used in a given context remains unanswered. There has been important work on assessing existing approaches and providing guidance for improvements [30, 47, 49, 50], but our searches did not identify any direct guidance on how to select the best approach for a given situation. Although our classification system was developed as a strategy to scrutinize the diversity of methodologies identified in the literature as value assessment frameworks, one can also rely on our 4-question analytical tool in order to develop novel ways to assess value in contexts hitherto unexplored.

## Conclusions

Three main lessons emerge from our literature review on value assessment approaches in health care. First, there is widespread interest amongst health care providers and funders in measuring value due to growing financial pressures. Second, the incremental cost-effectiveness ratio, the traditional value measurement tool, does not seem to meet the needs of decision-makers as demonstrated by the number of approaches being developed. And third, the definition of what is included in value and the methods and inputs involved in assessing performance have expanded significantly and continue to expand. Overall, this paper adds to the understanding of value assessment and should be a helpful guide as academics and decision makers seek to not only unpack the notion of value in health care but also move forward with initiatives to assess value in a given context.

Furthermore, our work reveals several important pathways of future research. First, the inconsistency found in the use of the notion of “value” shows the importance of a careful and robust epistemological reflection about this concept and its practical implications. Originally born within the realm of neoclassical economics with an intrinsic association with the notion of utility and its manifestation in price, value has gained other contours in the health economics literature. The widely cited work [1] by Michael Porter defines value as “health outcomes achieved per dollar spent”, which seems to place value as a maximand that fits well in a welfarist or extra-welfarist paradigm. More recently, the ISPOR’s task force [51] on value assessment presented a complex composite nature of value, although according to them only a few elements have been consistently addressed in the literature. Thus, not only a further reflection on the notion of value must be pursued by the health economics community, but also explicit statements about the underpinning theoretical notions of value should be encouraged by those who delve into empirical initiatives in this realm. Second, as many of the self-described value assessment frameworks did not present any explicit consideration of cost, it would be very important to understand how we can develop more appropriate tools that take into account the costs of health care interventions within each framework. That is related to the fact that the traditional cost-effectiveness approach does not seem to be currently fulfilling the needs of decision-makers and new manners to adequately consider cost may be also necessary. Lastly, just as our interpretive classification scheme, novel approaches to guide decision-makers and managers on how to choose and/or develop adequate practices to assess value of health care technologies need to be further investigated.

## Additional files

Additional file 1: Search strategy. (DOCX 30 kb)

Additional file 2: Full article data extraction tool. (DOCX 14 kb)

Additional file 3: Organizations identified in previous literature reviews as having signs of initiative around disinvestment and reassessment. (DOCX 14 kb)

## Data Availability

Not applicable.

## Abbreviations

AHRQ: Agency for Health Research and Quality
ASCO: American Society for Clinical Oncology
CADTH: Canadian Agency for Drugs and Technologies in Health
CEA: Cost-Effectiveness Analysis
CUA: Cost-Utility Analysis
DCE: Discrete Choice Experiment
EUnetHTA: European Network for Health Technology Assessment
HTA: Health Technology Assessment
HTAi: Health Technology Assessment International
ICER: Institute for Clinical and Economic Review
IHEA: International Health Economics Association
INAHTA: International Network of Agencies for Health Technology Assessment
ISPOR: International Society for Pharmacoeconomics and Outcomes Research
MCDA: Multi-Criteria Decision Analysis
RCT: Randomized Controlled Trial
VA: Veteran Affairs
