# Supplementary material for: Assessing value in health care: using an interpretive classification system to understand existing practices based on a systematic review
Source: BMC Health Serv Res. 2019 Aug 13;19:560. doi: 10.1186/s12913-019-4405-6 (PMC6693163; doi:10.1186/s12913-019-4405-6)
Supplement: Supplementary file 1 — Search strategy. (DOCX 30 kb) [file 12913_2019_4405_MOESM1_ESM.docx]

Additional file 1 – Search strategy


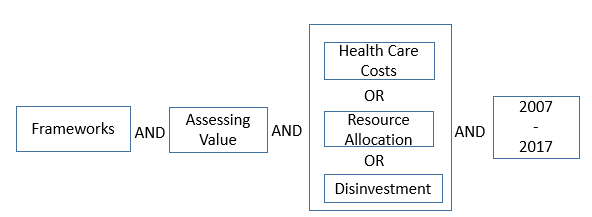


**Date:** Oct. 5, 2017

Database: Ovid MEDLINE(R) Epub Ahead of Print, In-Process & Other Non-Indexed Citations, Ovid MEDLINE(R) Daily, Ovid MEDLINE and Versions(R)

Search Strategy:

----------------------------------------------------------------

1 (framework or frameworks).tw,kw. (216370)

2 (tool or tools).tw,kw. (588414)

3 instrument?.mp. (195818)

4 case stud$.mp. (89760)

5 (approach or approaches).tw,kw. (1453071)

6 Organizational Case Studies/ (12027)

7 Concept Formation/ (11463)

8 concept formation.mp. (11788)

9 evidence informed deliberative processes.mp. (8)

10 multicriteria decision analysis.mp. (185)

11 multiple criteria decision analysis.mp. (46)

12 MCDA.mp. (413)

13 decision support techniques/ (18392)

14 decision analysis.mp. (4799)

15 GRADE evidence to decision.mp. [(EtD) frameworks] (20)

16 EVIDEM framework.mp. (15)

17 Health systems performance.mp. [WHO framework] (96)

18 health systems building blocks.mp. [WHO framework] (26)

19 wise list.mp. [essential drugs - Stockholm] (8)

20 "accountability for reasonableness".mp. [framework] (117)

21 "american society of clinical oncology".mp. [framework] (2369)

22 ASCO.mp. (2337)

23 "Institute for Clinical and Economic Review".mp. [framework] (24)

24 incremental cost-effectiveness ratio.mp. (4251)

25 ICER.mp. (3050)

26 European society for medical oncology.mp. [framework] (404)

27 ESMO.mp. (894)

28 technology assessment, biomedical/ or technology, high-cost/ (10746)

29 or/1-28 [Frameworks] (2359504)

30 Value-Based Purchasing/ (660)

31 (value adj5 (healthcare or health care)).mp. (2603)

32 value based purchasing.mp. (975)

33 (value adj6 (health or services)).mp. (9439)

34 relative value scales/ (1571)

35 (high value or low value).mp. (8605)

36 value purchasing.mp. (16)

37 or/30-36 [Assessing Value] (20723)

38 29 and 37 (4974)

39 health care costs/ or direct service costs/ or drug costs/ or employer health costs/ or hospital costs/ or health expenditures/ (74291)

40 health care costs/ or direct service costs/ or drug costs/ or hospital costs/ (58860)

41 health expenditures/ (17466)

42 Drug Costs/ (15019)

43 or/39-42 [Health Care Costs] (74291)

44 29 and 37 and 43 (271)

45 resource allocation/ (8091)

46 health care rationing/ (11495)

47 health priorities/ (10591)

48 Efficiency, Organizational/ (20763)

49 (priorit$ adj3 (setting or health)).mp. (20066)

50 "Cost Allocation"/ (2051)

51 (resource? adj3 (allocat$ or manag$)).mp. (30116)

52 or/45-51 [Resource Allocation] (77866)

53 29 and 37 and 52 (379)

54 (trade off adj5 (cost? or benefit?)).mp. (718)

55 commissioning.mp. (3764)

56 ration$.mp. (204395)

57 (restrict$ adj5 (treatment or price or prescrib$ or rate reduc$)).mp. (5126)

58 decision support techniques/ (18392)

59 (reinvest$ or re-invest$).mp. (8445)

60 (dis-invest$ or disinvest$).mp. (226)

61 (redeploy$ or re-deploy$).mp. (565)

62 (de-commission$ or decommission$).mp. (494)

63 (de-list$ or delist$).mp. (536)

64 (re-imburs$ or reimburs$).mp. (42462)

65 (re-negotiat$ or renegotiat$).mp. (401)

66 (de-adopt$ or deadopt$).mp. (51)

67 (reallocat$ or re-allocat$).mp. (2014)

68 "Cost Control"/mt [Methods] (3367)

69 "Cost Savings"/mt [Methods] (999)

70 (resource? adj3 releas$).mp. (210)

71 coverage.mp. (108715)

72 obsole$.mp. (4364)

73 (re-assess$ or reassess$).mp. (21235)

74 abandon$.mp. (19137)

75 low added value.mp. (11)

76 life cycle.mp. (37050)

77 or/54-76 [Disinvestment] (472159)

78 29 and 37 and 43 (271)

79 limit 78 to yr="2007 -Current" (192)

80 limit 79 to English language (188)

81 29 and 37 and 52 (379)

82 limit 81 to yr="2007 -Current" (250)

83 limit 82 to English language (242)

84 29 and 37 and 77 (723)

85 limit 84 to yr="2007 -Current" (540)

86 limit 85 to English language (524)

87 29 and **30** (133)

88 limit 87 to yr="2007 -Current" (133)

89 limit 88 to English language (132)

**Notes**

**Frameworks & Value & Health Costs**

78 29 and 37 and 43 (271)

79 limit 78 to yr="2007 -Current" (192)

80 limit 79 to English language (188)

**Frameworks & Value & Resource Allocation**

81 29 and 37 and 52 (379)

82 limit 81 to yr="2007 -Current" (250)

83 limit 82 to English language (242)

**Frameworks & Value & Disinvestment**

84 29 and 37 and 77 (723)

85 limit 84 to yr="2007 -Current" (540)

86 limit 85 to English language (524)

**Frameworks & Value**

87 29 and **30** (133)

88 limit 87 to yr="2007 -Current" (133)

89 limit 88 to English language (132)
