# Supplementary material for: Assessing value in health care: using an interpretive classification system to understand existing practices based on a systematic review
Source: BMC Health Serv Res. 2019 Aug 13;19:560. doi: 10.1186/s12913-019-4405-6 (PMC6693163; doi:10.1186/s12913-019-4405-6)
Supplement: Supplementary file 2 — Full article data extraction tool. (DOCX 14 kb) [file 12913_2019_4405_MOESM2_ESM.docx]

Additional file 2 – Full article data extraction tool

- What is the name of the value assessment tool/ approach/ framework/ process?
- What country/ region was the tool/ approach utilized, who used it, and in what context (i.e., what was the purpose)?
- Is the tool/ approach novel or has it been used previously?
- Describe the tool/ approach:
- How does the tool define value?
- Is the tool disease-specific?
- What is the result of the application of the tool (e.g., a score)?
- How is the result arrived at?
- Does the tool specifically include patients’ feedback?
- Was the implementation formally evaluated and, if so, what were the findings of the evaluation?
