# Supplementary material for: Assessing value in health care: using an interpretive classification system to understand existing practices based on a systematic review
Source: BMC Health Serv Res. 2019 Aug 13;19:560. doi: 10.1186/s12913-019-4405-6 (PMC6693163; doi:10.1186/s12913-019-4405-6)
Supplement: Supplementary file 3 — Organizations identified in previous literature reviews as having signs of initiative around disinvestment and reassessment. (DOCX 14 kb) [file 12913_2019_4405_MOESM3_ESM.docx]

Additional file 3 – Organizations identified in previous literature reviews as having signs of initiative around disinvestment and reassessment.

| Australia | - Medical Services Advisory Committee (MSCA)  - Health Policy Advisory Committee for Technology (HealthPACT)  - Pharmaceutical Benefits Advisory Committee (PBAC) |
| --- | --- |
| Norway | - Norwegian Council for Quality Improvement and Priority Setting in Health Care |
| Scotland | - Scottish Health Technologies Group (SHTG) |
| Spain | - Basque Office for HTA (OSTEBA)  - Galician Agency for HTA (Avalia-T) |
| Sweden | - Swedish Council on Technology Assessment in Health Care (SBU) |
